# Detoxication of Citrinin with Kojic Acid by the Formation of the Citrinin-Kojic Acid Adduct, and the Enhancement of Kojic Acid Production by Citrinin via Oxidative Stress in *Aspergillus parasiticus*

**DOI:** 10.3390/jof9010051

**Published:** 2022-12-29

**Authors:** Masayuki Ichinomiya, Ayaka Kawamoto, Takahiro Yamaguchi, Keiko Iwashita, Hitoshi Nagashima, Hidemi Hatabayashi, Hiromitsu Nakajima, Kimiko Yabe

**Affiliations:** 1 Institute of Food Research, National Agriculture and Food Research Organization (NARO), 2-1-12 Kannon-dai, Tsukuba-shi, Ibaraki 305-8642, Japan; 2 Faculty of Agriculture, Tottori University, Koyama, Tottori 680-8553, Japan; 3 Department of Applied Chemistry and Food Science, Fukui University of Technology, 3-6-1 Gakuen, Fukui-shi, Fukui 910-8505, Japan

**Keywords:** α-tocopherol, *Aspergillus oryzae*, *Aspergillus parasiticus*, endoplasmic reticulum stress, oxidation stress, mycotoxin, *Penicillium citrinum*, reactive oxygen species

## Abstract

Our previous work showed that citrinin (CTN) produced bay *Penicillium citrinum* inhibited the production of aflatoxin by *Aspergillus parasiticus.* We also reported that CTN was non-enzymatically converted to a novel CTN-KA adduct with kojic acid (KA) in aqueous condition. We herein observed that unlike CTN, the CTN-KA adduct does not show antimicrobial activity against *Escherichia coli* or *Bacillus subtilis* or any cytotoxic effect on HeLa cells, suggesting that CTN was detoxified by KA by the formation of the CTN-KA adduct. To examine the function of KA production by fungi, we isolated *A. parasiticus* mutants with impaired KA production. When the mutants were incubated in either liquid or agar medium supplemented with CTN, they were more susceptible to CTN than the wild KA-producing strain. The same results were obtained when we used the *A. oryzae* KA-producing strain RIB40 and KA-non-producing strains. When KA was added to the CTN-containing agar medium, the inhibition of growth by CTN was remarkably mitigated, suggesting that the production of KA protected the fungal growth from CTN’s toxicity. We also observed that CTN enhanced the production of KA by *A. parasiticus* as well as *A. oryzae* strains. Reverse transcription-PCR showed that CTN enhanced the expression of KA biosynthetic genes (*kojA*, *kojR*, and *kojT)* of *A. parasiticus*. However, the enhancement of KA production with CTN was repressed by the addition of α-tocopherol or butylated hydroxy anisole, suggesting that KA production is enhanced by oxidative stress via the formation of reactive oxygen species caused by CTN. In contrast, α−tocopherol did not affect inhibition of AF production as well as fungal growth by CTN, suggesting that the regulation of these inhibitions with CTN might be different from that of KA production. We propose a regulation scheme of CTN for each of KA production, AF production, and fungal growth in *A. parasiticus*.

## 1. Introduction

Mycotoxins are toxic secondary metabolites produced by fungi. The contamination of crops, food, and feed with various types of mycotoxins causes serious health problems in animals and humans, as well as financial and other losses [1,2]. Most mycotoxigenic fungi belong to three genera (*Aspergillus*, *Penicillium*, and *Fusarium*), and they are widely distributed in many crops, plants, food products, and supplements. Various types of mycotoxins have been reported, and among them the aflatoxins (AFs) are highly toxic, carcinogenic, teratogenic, and mutagenic substances, causing acute hepatoxicity or liver cancer. AFs are produced primarily by certain strains of *Aspergillus flavus* Link and *Aspergillus parasiticus* Speare, which are relatively abundant in agricultural soils. Although aflatoxigenic fungi have been observed to be able to produce eight types of AFs, they produce mainly aflatoxins B_1_ (AFB_1_), B_2_ (AFB_2_), G_1_ (AFG_1_), and G_2_ (AFG_2_). Among these, AFB_1_ is the most toxic and carcinogenic substance and is relatively abundantly produced, and it is classified in group 1 (human carcinogens) by the International Agency for Research on Cancer [3].

Citrinin (CTN) is a mycotoxin produced by various fungal strains belonging to mainly the genera *Penicillium* and *Monascus*. CTN contamination has been widely detected in cereals, feed, and supplements [4]. CTN is a nephrotoxic and hepatotoxic substance to animals and is an antibiotic, antifungal, anti-bacteriophage, anti-sarcoma, anti-protozoa and anti-animal cells substance [5]. Much research has been conducted to develop strategies to combat and control AF and CTN contamination, and the inhibition of the production of these mycotoxins with various types of microorganisms such as bacteria, yeasts, and fungi [6,7,8] and plant extracts [9] has been suggested.

Kojic acid is a secondary metabolite produced by many kinds of *Aspergillus* species such as *A. oryzae*, *A. sojae*, and *A. awamori*. KA is a by-product in the fermentation process for the production of many Asian and Japanese food products [10]. It is well known as a tyrosinase inhibitor and is used in food and cosmetics to preserve or change colors of substances. It forms a bright red complex with ferric ions [11].

**Figure 1:**
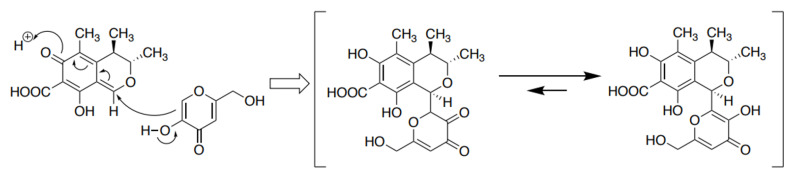
Formation of the citrinin-kojic acid (CTN-KA) adduct. In the presence of KA in an aqueous condition, CTN is non-enzymatically converted to form the CTN-KA adduct [12].

Regarding the inhibition of AF contamination with microorganisms, in our recent work we observed that *Penicillium citrinum* strains isolated from the environment inhibited the production of AF by *A. parasiticus* and the fungal growth of this strain; moreover, the inhibitory substance produced by *P. citrinum* was confirmed to be CTN [12]. We also observed that CTN in the culture medium of *A. parasiticus* was converted to a novel compound that we named ‘CTN-KA adduct’: (1*R*,3*S*,4*R*)-3,4-dihydro-6,8-dihydroxy-1- (3-hydroxy-6-(hydroxymethyl)-4-oxo-4*H*-pyran-2-yl)-3,4,5-trimethyl-1*H*-isochromene-7-carboxylic acid (Figure 1). This conversion occurred non-enzymatically in aqueous conditions at mild temperatures, i.e., 28 °C, 37 °C, and 50 °C. We also confirmed that unlike CTN, the CTN-KA adduct did not inhibit the production of AF, suggesting that the CTN-KA adduct may be a detoxicated form of CTN.

We conducted the present study to further examine the toxicity of the CTN-KA adduct toward bacteria (*Escherichia coli* and *Bacillus subtilis*) and HeLa (human epithelial) cells. The results confirmed that unlike CTN, the CTN-KA adduct did not show toxicity to these bacteria and cells, suggesting that the CTN-KA adduct is indeed a detoxication form of CTN. To examine CTN-KA adduct formation in fungi, we isolated various mutants with impaired KA production from *A. parasiticus* and investigated their sensitivities to CTN. The mutants were observed to be more susceptible to CTN than the wildtype, suggesting that KA functions as a protector from CTN attack. We also investigated the effects of CTN on the productions of KA and AFs and the fungal growth of KA-producing fungi: the production of KA by *A. parasiticus* was enhanced by CTN, and this enhancement of KA was alleviated with α-tocopherol, suggesting that reactive oxygen species (ROS) may be involved in the production of KA by CTN. We propose a mechanism of the effects of CTN on *A. parasiticus* and *A. oryzae.*

## 2. Materials and Methods

### 2.1. Microorganisms

Fungi used in this work are shown in Table 1. An *E. coli* strain, *B. subtilis* strain, and HeLa cells (RIKEN Cell Bank, Tsukuba, Japan) were also used for the determination of toxicities of CTN, KA, and the CTN-KA adduct.

**Table 1:** Fungi used in this work.

Fungi	Metabolism	Phenotype	Ref. No.
*A. parasiticus* SYS-4	AF^+^, KA^+^	*A. parasiticus* wild strain (=NRRL2999)	[13]
*A. parasiticus* NFRI-95	NA^+^, AFΔ ^(1)^, KA^+^	a NA-accumulating mutant derived from *A. parasiticus* SYS-4 by UV irradiation	[13]
*A. parasiticus* NIAH-26	AF^−^, KA^+^	a mutant of *A. parasiticus* SYS-4 that produces neither AFs nor any pigmented precursors of AFs	[13]
*A. parasiticus* 11AP, 34C8, 48B11, 34C8, and 59A6	AF^+^, KAΔ ^(1)^ or AF^+^, KA^−^	The isolated mutants with impaired KA productivity from SYS-4 by UV irradiation	This work
*A. oryzae* RIB40	AF^−^, KA^+^	*A. oryzae* wild strain (NRIB, Japan)	
*A. oryzae* RIB143, RIB430, and RIB1025	AF^−^, KA^−^	*A. oryzae* KA-non-producing strains (NRIB)	
*Penicillium citrinum* NFRI-MI190	CTN^+^	The fungus isolated from soil as an inhibitory fungus to aflatoxin production by *A. parasiticus*	[12]

(1) “Δ” means a leaky mutant of the metabolite.

### 2.2. Metabolites

Citrinin (Sigma-Aldrich, St. Louis, MO, USA) or CTN sample purified from the culture medium of *P. citrinum* NFRI-MI190 was used. Culture filtrate of the NFRI-MI190 strain was also used when specified. KA (Tokyo Chemical Industry, Japan) and KA purified from the culture medium of *A. parasiticus* NFRI-26 were used. The CTN-KA adduct was prepared after the incubation of KA and CTN in buffered water (10 mM ammonium acetate buffer pH 7.0 or 50 mM K-phosphate buffer pH 7.5) at 50 °C for 6 h, and then purified by high-performance liquid chromatography (HPLC).

### 2.3. Media

Luria-Bertani (LB) broth (1% tryptone, 0.5% yeast extract, 1% NaCl) was used for growing *E. coli* and *B. subtilis*. For the culturing of HeLa cells, we used Dulbecco’s modified Eagle medium (DMEM) (Nissui Pharmaceutical Co., Tokyo, Japan) supplemented with 20% heat-inactivated fetal bovine serum (JRH Biosciences, Lenexa, KS, USA), 100 U mL^−1^ penicillin, and 100 μg mL^−1^ streptomycin.

GY agar medium (2% glucose, 0.5% yeast extract, 2% agar) was used for the visual agar plate assay and the microtiter agar plate assay. Liquid media of YES (2% yeast extract, 20% sucrose), GY (2% glucose, 0.5% yeast extract), and YEP (2% peptone, 0.5% yeast extract) were used for the tip culture method. For the selection of KA-non-producing mutants from *A. parasiticus* SYS-4 strain, we used KM2 agar medium (0.25% yeast extract, 1.0% KH_2_PO_4_, 0.05% MgSO_4_·7H_2_O, 0.05% KCl, 0.001% FeSO_4_, 10% glucose, 2.0% agar, pH 6.0) (Japan Patent Tokuhyo 2002-533133 (2002.10.08)). For the examination of the effect of *P. citrinum* on the growth of various *Aspergillus* strains, 20 μL of 200-fold-diluted dichlorvos (DV) [14] with methanol was spread onto a GY agar plate (9 cm. dia.).

### 2.4. Effect of Each Metabolite on Bacterial Growth

For the examination of the effect of each metabolite on bacterial growth, we dispensed 100 μL of LB medium into a 96-well plate, and 3 μL of acetone solution containing each of CTN, CTN-KA adduct, and KA was, respectively added and mixed. After *E. coli* and *B. subtilis* were precultured at 30 °C for 24 h and 48 h, respectively, either preculture (10 μL) was added to 100 μL of LB medium in each well and then shake-cultured at 30 °C for 24 h. We estimated the bacterial growth by monitoring the absorption at 660 nm using a microplate reader (Sunrise Rainbow RC, Fujifilm Wako Pure Chemical Co., Osaka, Japan). Experiments were done in triplicate.

### 2.5. Effect of Each Metabolite on Growth of HeLa Cells

To examine cytotoxicity of the metabolites, Cell Counting Kit-8 (DOJINDO CK04, Kumamoto, Japan) was used according to the company instruction. The cytotoxicity test was based on a mitochondrial enzyme activity. HeLa cells (5 × 10^3^ cells) were cultured in 100 μL of Dulbecco’s Modified Eagle’s Medium (Nissui Pharmaceutical Co., Ltd., Tokyo, Japan) containing 10% fetal calf serum, which had been supplemented without or with 0.48 mM each of CTN, KA, and CTN-KA adduct, in each well of 96-well microplate in a CO_2_ incubator for 24 h. Ten microliter of CCK-8 solution was then added to each well and cultured for another 2 h. Absorbance at 450 nm in each well were measured by a Microplate reader (Sunrise Rainbow RC, Wako Pure Chemical Industries, Ltd., Osaka, Japan). The experiment was done in sextuplicate. Morphology of HeLa cells in each well was also observed using Microscopy.

### 2.6. Tip Culture Method

To examine the production of fungal metabolites as well as fungal growth, we used the tip culture method in which a Pipetman^®^ tip (P1000) stuffed with quart wool was used as a culture vessel [13]; 5 μL of spore suspension of each fungus was then inoculated into 200 μL of liquid medium supplemented either without or with CTN in tip culture. After 2, 3 or 4 days of incubation at 28 °C, the mycelial mat and culture medium were separated by centrifugation. For the detection of metabolites in the medium, 10 μL of the medium was analyzed by thin-layer chromatography (TLC) using silica gel plates (Silica Gel 60; Merck, Darmstadt, Germany) and each of developing solutions: solution A, chloroform-ethyl acetate-90% formic acid (6:3:1, *v*/*v*/*v*) [15]; solution B, hexane-ethyl acetate-acetic acid (50:50:1, *v/v/v*); solution C, chloroform-acetone-isopropanol (85:15:20, *v/v/v*); and solution D, toluene-ethyl acetate-acetic acid (6:3:1, *v/v/v*). The TLC plate was observed under long-wavelength (365 nm) UV light. For the detection of KA, the TLC plate was sprayed with 1% FeCl_3_ in 0.1 M HCl aqueous solution [11].

### 2.7. Isolation of A. Parasiticus Mutants with Impaired KA Production

Conidia of *A. parasiticus* SYS-4 strain were suspended in 0.05% Tween 80 at the concentration of 2.0 × 10^6^ mL^−1^ and then irradiated with UV light (254 nm) for 105 s (the viability was 5.5%), followed by 30 min stirring without irradiation. The suspension was diluted and spread on KM2 solid medium supplemented with 0.03% Triton X-100. After incubation at 28 °C for 3 days, conidia from each colony were picked up with a toothpick and inoculated into 100 μL of KM2 liquid medium in each well of a 96-well microtiter plate. After incubation at 28 °C for 4 days, the KA production of each colony was examined after the addition of 40 μL/well of 1% FeCl_3_/0.1 M HCl and the visual inspection of the development of a dark-red color. Among about 7000 colonies, 96 colonies lost the dark-red color, indicating that these fungi were likely desired candidates.

For the second screening, conidia of each colony were spread on KM2 agar medium supplemented with 0.03% Triton X-100. Based on the inspection of color development, 29 mutants were selected. These mutants were then cultured by the tip culture method, and each resulting medium was analyzed by two-dimensional TLC analyses using a first solvent, i.e., chloroform-acetone-isopropanol (85:15:20, *v/v/v*) followed by a second solvent, i.e., toluene-ethanol-formic acid (5:4:1, *v/v/v*). The visualization of the production of KA was achieved by spraying the TLC plate with 1% FeCl_3_ in 0.1 M HCl solution [11]. Six mutants were finally obtained as the mutants with remarkably decreased or zero KA productivity.

### 2.8. Measurement of KA Concentration in the Fungal Media

The KA concentration in the fungal media was measured by Bentley’s method [11]. After tip culture, the resulting culture medium was diluted by 20 times with water. A 10-μL aliquot of the diluted solution was added to 90 μL of 1% FeCl_3_ in 0.1 M HCl solution and mixed by a vortex mixer for 20 s. The absorption of the solution at 505 nm was monitored, and we estimated the concentrations of KA by applying a calibration curve created with an authentic KA standard.

### 2.9. Co-Culture of Two Different Fungi

For the detection of the effect of *P. citrinum* NFRI-MI190 on the production of AF as well as the effect on the fungal growth of various *A. parasiticus* and *A oryzae* strains, we inoculated a spore suspension of MI190 and each of the *Aspergillus* strains at the distance of 3 cm on the same GY plate spread with DV and then incubated the plate for 7 days. The resulting plate was placed up-side down, and then 0.25 mL of ammonia solution (FUJIFILM Wako Chem. Co., Osaka, Japan) was added to the lid of the plate. The colonies were observed from the underside of the plate.

### 2.10. RT-PCR

*A. parasiticus* NIAH-26 was cultured by the tip culture method using 200 μL of YES medium supplemented with or without 0.63 mM CTN at 28 °C for 3 days. The mycelial mat was separated from the culture medium by centrifugation and then put into a 2-mL microtube containing zirconia beads and 300 μL of triazole (Thermo Fisher Scientific, Waltham, MA, USA). The entire tube was shaken by using a FastPrep FP100A instrument (Q-BIO gene; BIO 101, Vista, CA, USA) at 6.5 m/s for 45 s. After centrifugation at 12,000 rpm at 4 °C for 10 min, 200 μL of the supernatant was transferred to a new tube, and the total RNA in the solution was prepared by using the Direct-zol RNA MiniPrep kit (ZYMO Research, Irvine, CA, USA) according to the manufacturer’s instructions. cDNA was then prepared from the resulting RNA fraction by using iScript RT Supermix (Bio-Rad Laboratories, Hercules, CA, USA) according to the manufacturer’s instructions. The polymerase chain reaction (PCR) was conducted using the appropriate primers for each of *kojA*, *kojR*, *kojT* [16] and 28S rDNA genes: for *kojA,* kojAF [GCCACCGAGTTTCTTTGTTC] and kojAR [CCACTCCCGAACCACACC]; for *kojR* gene, kojRF [ATCCGGAACTGGTGTCTTTG] and kojRR [GAACCTGGGTGTCCAAGAAC]; for *kojT* gene, kojTF [GAGAGTGACCGTTCCTGGAGT] and kojTR [GGAACCCGAATAGGACAATG]; and for 28S rDNA gene, LROR [ACCCGCTGAACTTAAGC] and LR5 [TCCTGAGGGAAACTTCG].

## 3. Results

### 3.1. Toxicity of the CTN-KA Adduct to Bacteria as Well as HeLa Cells

**Figure 2:**
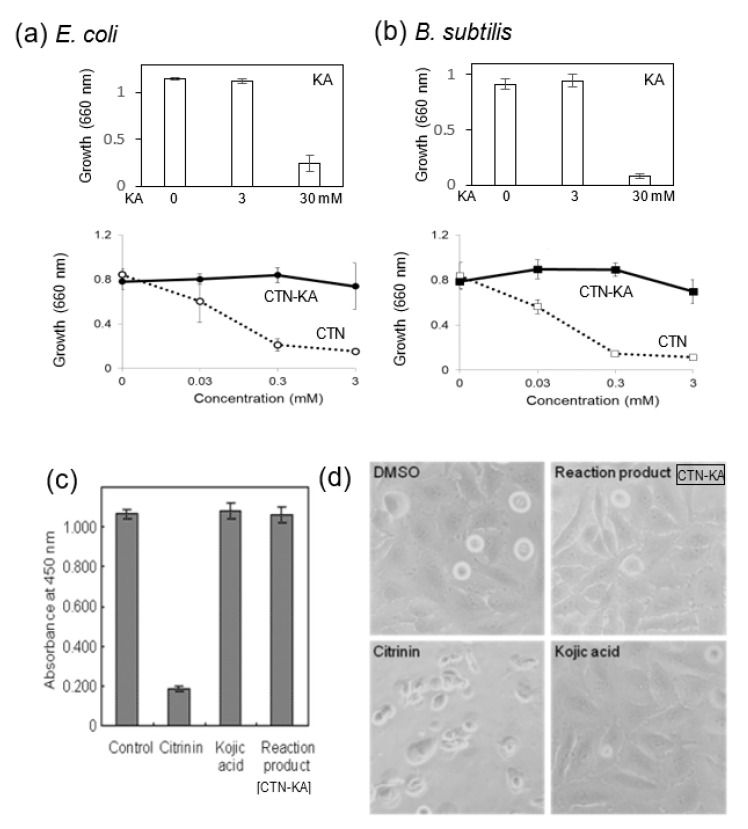
The toxicities of CTN, the CTN-KA adduct, and KA on bacteria and mammalian cells. The toxic effects on *E. coli* (**a**) and *B. subtilis* (**b**). Both bacteria were incubated in LB broth supplemented with 3 mM or 30 mM KA in triplicate and then analyzed growths of the bacteria (upper panels). The bacteria were incubated with either CTN or CTN-KA adduct and then the resulting growths were analyzed (lower panels). (**c**) The toxic effects on HeLa cells. HeLa cells were cultured in media supplemented without (control) or with 0.48 mM of CTN, the CTN-KA adduct, and KA, respectively for 24 h., and then CCK-8 solution was added and cultured for 2 h. Absorbance at 450 nm at each well was measured. High absorbance indicated better growth of the cells. Experiments were done in sextuplicate. (**d**) Phenotypes of the resulting HeLa cells. Reaction product in a photo showed the CTN-KA adduct.

CTN is known to have antibacterial activity. We investigated the effects of the CTN-KA adduct as well as those of CTN on the growth of *E. coli* and *B. subtilis*. We first observed that 3 mM KA did not affect the growth of either of these bacteria, whereas 30 mM KA remarkably inhibited both growths (Figure 2a,b, upper panels). When various concentrations of CTN were added to the culture medium, the growth of *E. coli* was severely inhibited at ≥0.3 mM, whereas the same concentrations of the CTN-KA adduct did not inhibit the bacterial growth (Figure 2a, lower). Similar results were obtained when *B. subtilis* was used (Figure 2b, lower). These results indicated that the toxicity of CTN was alleviated or lost due to a structural change of the CTN-KA adduct in these bacteria.

We also used HeLa cells to investigate the toxic effects of CTN, KA, and the CTN-KA adduct. HeLa cells were cultured in the presence of the CTN-KA adduct, CTN, and KA (0.48 mM each) for 24 h. and then treated with CCK-8 solution. The addition of CTN to the cells decreased absorption at 450 nm to <10% of that obtained in the control experiment without any substance, indicating that CTN inhibited the growth of the HeLa cells at this concentration (Figure 2c). In contrast, the addition of the same concentration of either the CTN-KA adduct or KA did not exert any adverse effect on the growth of HeLa cells. The morphological observation of the cells by microscopy revealed that CTN caused shrinkage as well as scattering of the cells, suggesting the CTN had toxic effects on the HeLa cells (Figure 2d). The addition of the CTN-KA adduct as well as that of KA did not result in a significantly different cell phenotype. These results indicated that the cytotoxicity of CTN to HeLa cells was detoxicated with KA by the formation of the CTN-KA adduct.

### 3.2. Effect of CTN on KA-Producing or KA-Non-Producing Fungi by the Tip Culture Method 

**Figure 3:**
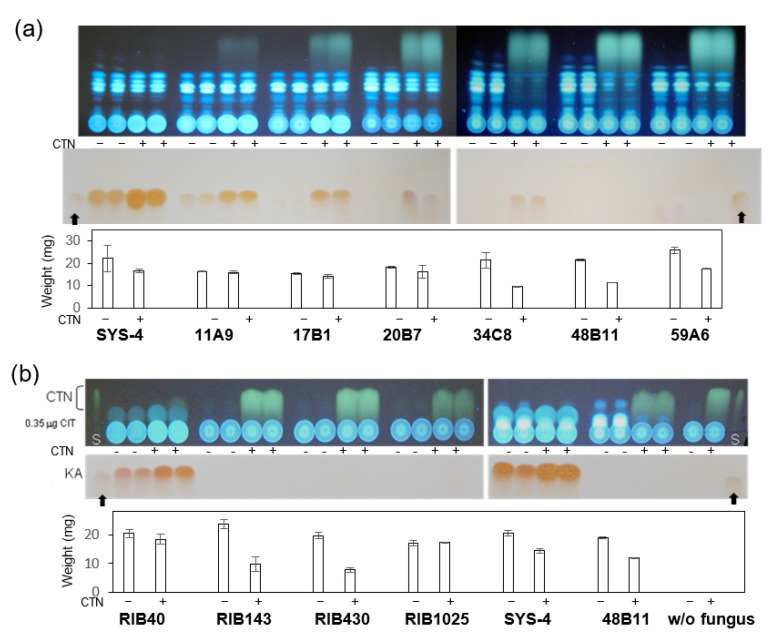
The production of AF and KA by various fungi. (**a**) The effect of CTN on *A. parasiticus* SYS-4 and its mutants. Aflatoxigenic SYS-4 strain and its mutants with reduced or impaired KA production (11A9, 17B1, 20B7, 34C8, 48B11, and 59A6) were incubated in the absence or the presence of 0.1 mg mL^−1^ (0.4 mM) CTN for 4 days by the tip culture method, in duplicate. After centrifugation, each culture medium (10 μL) was analyzed by TLC using the developing solution A. AFs and CTN were detected by observation under UV light (**upper** panel). KA was observed by the spraying of 1% FeCl_3_ in 0.1 M HCl solution onto the TLC plate followed by heating at 80 °C for 20 min (**middle** panel). Ten microliters of 10 mM KA standard solution ware also analyzed as markers (thick arrow). The weight of the mycelia that remained in the tip was measured, and the mean and differences of the weights were shown for each fungus (**lower** panel). (**b**) The effect of CTN on *A. oryzae* and *A. parasiticus* strains. *A. oryzae* RIB 40, other non-KA producing *A. oryzae* strains (RIB143, RIB430, and RIB1025), and *A. parasiticus* SYS-4 as well as 48B11 were incubated without or with 0.1 mg mL^−1^ CTN for 4 days. The metabolites in the resulting culture medium were analyzed by TLC using either the developing solution B (**upper** panel) or solution C (**middle**). The weight of the mycelia that remained in the tip was shown (**lower**). Lane S: 0.35 μg CTN (**upper**).

To study the function of KA production in fungi, we isolated mutants with impaired KA production by the UV irradiation of *A. parasiticus* SYS-4. After SYS-4 and the resulting mutants were, respectively cultured in the medium without or with 0.1 mg mL^−1^ CTN for 4 days, metabolites in each culture medium were analyzed by TLC (Figure 3). The AF productivity of SYS-4 had already recovered after 4-day incubation, whereas the CTN first added completely disappeared (Figure 3a, upper panel); this is the same result obtained in our earlier study [12]. The KA productivity of SYS-4 was remarkably enhanced by CTN, suggesting that the production of KA was induced by CTN (Figure 3a, lower panel). The KA production in two ‘leaky’ mutants, 11A9 and 17E1, was also enhanced by CTN, although a small amount of CTN still remained even after 4 days. In contrast, a partial inhibition or complete inhibition of AF production was observed in the other five mutants, as relatively large amounts of CTN remained after 4-day incubation. Four mutants, i.e., 20B7, 34C8, 48B11, and 59A6, were first isolated as KA-non-producers in YES medium, and the production of KA was newly observed by the addition of CTN in the 20B7 and 34C8 mutants, in contrast, the other mutants did not produce KA even with the addition of CTN. Significant amounts of CTN remained in these four mutants.

To test this possibility, we investigated whether CTN affects the growth of other fungi, i.e., *A. oryzae* RIB40 (a wild strain) and three KA-non-producing strains: RIB143, RIB430, and RIB1025 (Figure 3b). We also repeated the experiments using *A. parasiticus* SYS-4 and 48B11 mutant. Although CTN inhibited the growth of all fungi, the inhibition rates of the KA-non-producing *A. oryzae* strains other than RIB1025 were higher in the KA-non-producing mutants (~40% of weight in the absence of CTN) compared to the KA-producing wild strains (~93%). The growth rate of *A. parasiticus* SYS-4 was 69%, and that of the 48B11 mutant was 63%. The same experiments showed that CTN enhanced the KA production of RIB40 as well as SYS-4, whereas other strains did not produce KA irrespective of the addition of CTN, indicating that the absence of KA production caused fungi to be more susceptible to a CTN attack. It was also confirmed that first-added CTN mostly remained even after 4-day incubation, suggesting that CTN could not be consumed with KA.

### 3.3. Effects of CTN on KA Producers and KA Non-Producers in Agar Medium

**Figure 4:**
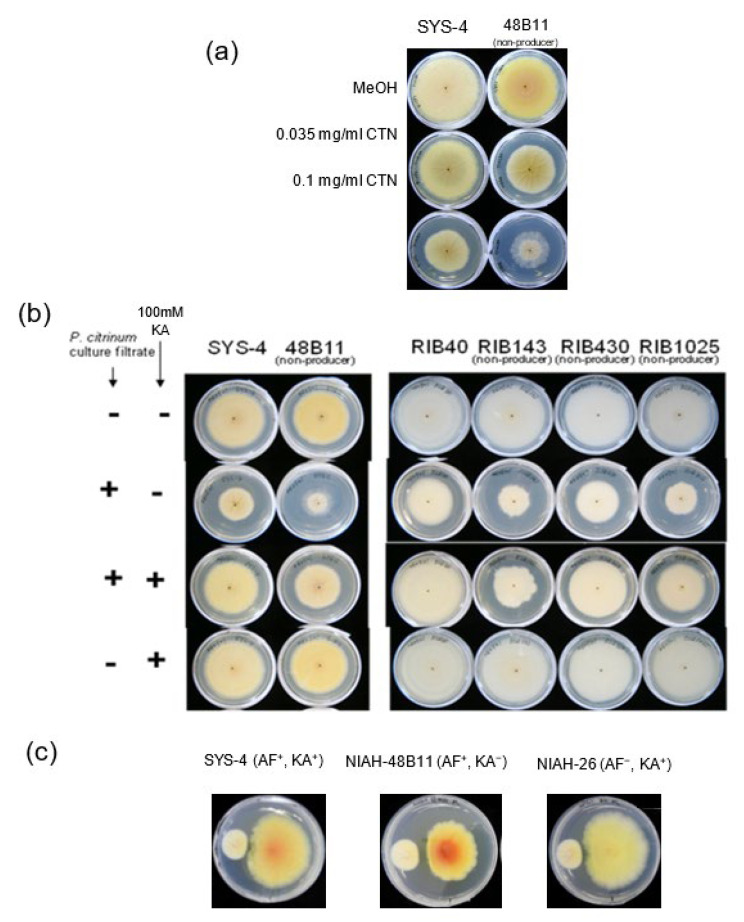
The CTN sensitivity of KA producers or non-producers. (**a**) *A. parasiticus* NIAH-59A6 (KA non-produced: KA^−^), NIAH-48B11 (KA^−^), and SYS-4 (KA^+^) were, respectively, incubated in GY agar medium (7 mL in a 6-cm-dia. plate) containing 0, 0.035 mg mL^−1^ (0.14 mM), or 0.1 mg mL^−1^ (0.42 mM) of CTN for 4 days. In order to facilitate the comparison with the results in panel (**b**), the left and right sides of the photos are presented in reverse. (**b**) *A. parasiticus* SYS-4 (KA^+^) or its mutant, NIAH-48B11 (KA^−^), was incubated in a GY agar plate supplemented (10-fold dilution) with the culture filtrate *P. citrinum* NFRI-190 and/or 100 mM KA for 4 days (**left** panel). *A. oryzae* RIB40 (KA^+^), RIB143 (KA^−^), RIB430 (KA^−^), and RIB1025 (KA^−^) were also cultured in the same media for 5 days (**right**). (**c**) The effect of *P. citrinum* NFRI-190 on the fungal growth of the strains SYS-4, NIAH-48B11 (KA^−^), and NIAH-26. Each strain was incubated at the center of the plate, and *P. citrinum* NFRI-190 was inoculated at 3 cm from the center onto GY plates spread with DV. After 7-day incubation, the colonies were observed from the underside of the plate.

We analyzed the effects of CTN on the fungal growth by using agar media (Figure 4). The KA-non-producing strain 48B11 and wild SYS-4 strain were, respectively inoculated onto GY agar medium supplemented with 0.035 mg mL^−1^ or 0.1 mg mL^−1^ CTN and then cultured for 4 days. At both concentrations, the growth of 48B11 was more severely inhibited compared to the growth of SYS-4, suggesting that 48B11 was more susceptible to CTN, probably due to its lack of KA productivity (Figure 4a).

The same fungi were inoculated onto GY agar medium supplemented with a 10% volume of the culture filtrate of *P. citrinum* containing CTN (Figure 4b). Although their colonial sizes remarkably decreased, especially in the 48B11 mutant (Figure 4b, left), the growth of each strain was remarkably recovered by the co-addition of 100 mM KA to the same medium. The simple addition of KA did not affect the growth of either strain. The same experiments were done using *A. oryzae* RIB40, RIB143, RIB430, and RIB1025 (Figure 4b, right). Although the addition of the culture filtrate containing CTN caused the inhibition of the growth of all these fungal strains, the KA-non-producers appeared to be more susceptible to CTN. The co-addition of KA to the medium also resulted in the significant recovery of all colonial sizes. Although susceptibility of RIB1025 to CTN was not remarkable by the tip culture (Figure 3b), it was confirmed using culture using agar medium (Figure 4b). These results demonstrated that fungal KA production as well as exogenous supplementation of KA mitigated the toxicity of CTN.

For an investigation of the in vivo interaction between *P. citrinum* and *Aspergillus* strains, the CTN-producing strain MI-190 and the SYS-4 (KA^+^), 48B11 (KA^−^), and NFRI-26 (KA^+^) strains were, respectively inoculated at a distance of 3 cm on GY medium spread with DV and then cultured for 7 days (Figure 4c). The use of MI-190 inhibited the growth of both SYS-4 and NIAH-26 only at the side of MI-190. In contrast, the use of MI-190 more remarkably inhibited the entire colony of 48B11, and the resulting colony became much smaller; the longest diameter as well as the shortest diameter was approx. 77% of those of SYS-4. These results indicated that the KA-non-producing strain 48B11 was more susceptible to CTN produced by MI-190. The orange and yellow colors of the SYS-4 and 48B11 plates corresponded to an accumulation of AF precursors, as it was reported that DV inhibits a certain enzyme in the biosynthesis of AF [14]; this suggests that AF production appears to be unaffected by CTN in this condition. The colony of NIAH-26 was white because this mutant lacks AF productivity [13].

### 3.4. Effects of CTN and α-Tocopherol on KA Production by Fungi

**Figure 5:**
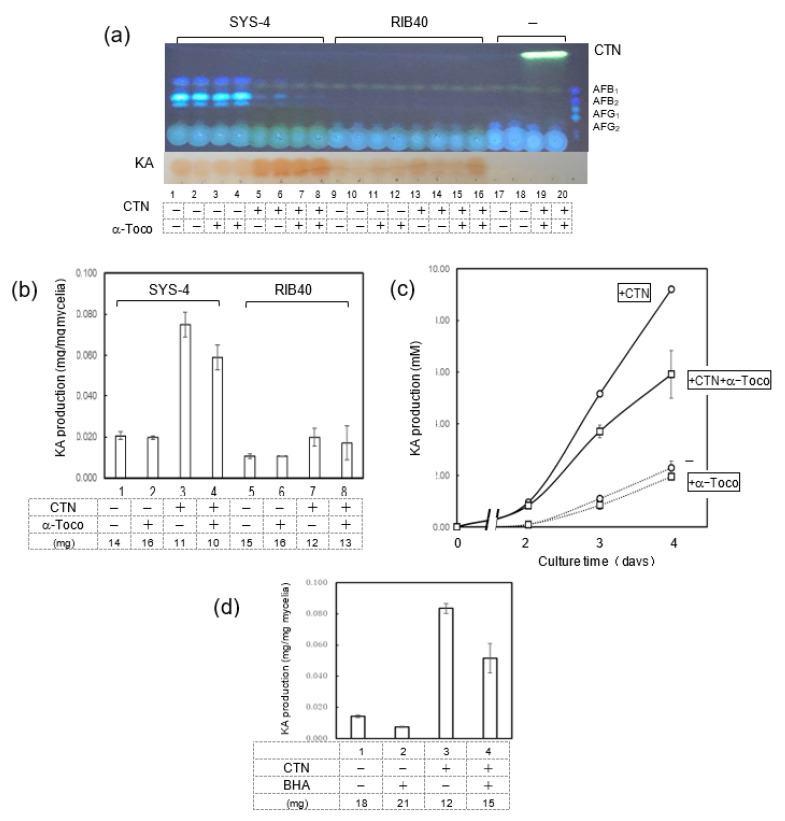
The enhancement of KA production by CTN and its inhibition by α-tocopherol. (**a**) *A. parasiticus* SYS-4 and *A. oryzae* RIB40 were each cultured in YES medium containing either/both of 0.63 mM CTN and 0.1 mM α-tocopherol (α-T) by tip culture method for 3 days. The resulting culture media were analyzed by TLC using solution D. The KA produced was observed by spraying 1% FeCl_3_ in 0.1 M HCl solution. (**b**) The KA contents of the culture media described in (**a**) were measured by the Bentley method. The weights of the mycelia are also shown. (**c**) The time course of the production of KA by *A. parasiticus* SYS-4 was analyzed in the absence (dashed line) and presence (solid line) of CTN and in the absence (open circles) and presence (open squares) of α-tocopherol. (**d**) *A. parasiticus* NIAH-26 was cultured in the absence and presence of 0.63 mM CTN or 0.2 mM BHA by tip culture for 3 days. Mycelial weights are also shown.

To examine the enhancement of KA production with CTN, we investigated the effect of α-tocopherol, an antioxidant reagent, on the production of KA by using the tip culture method. SYS-4 and RIB-40 strains were incubated with either or both CTN and α-tocopherol for 3 days in tip culture and then analyzed by TLC (Figure 5a). In addition, after the culture medium of the tip culture was mixed with FeCl_3_/0.1 M HCl, the absorption was monitored at 505 nm (Figure 5b). CTN enhanced the SYS-4 strain’s production of KA, and the amount of KA produced was partially decreased by the addition of α-tocopherol. The addition of CTN resulted in a remarkable decrease of mycelial weights, and this weight decrease was not recovered with the addition of α-tocopherol. Similar results were obtained when *A. oryzae* RIB-40 was used, although its KA productivity was remarkably small compared to that of SYS-4. The similar effects of CTN and α-tocopherol on the production of KA by strain SYS-4 were confirmed when SYS-4 was cultured for 2–4 days in the same conditions (Figure 5c).

When butylated hydroxyanisole (BHA), another antioxidant reagent, was used instead of α-tocopherol in the same experiments but with the use of strain NIAH26, the production of KA by CTN was significantly decreased (Figure 5d). These results demonstrated that the enhancement of KA production by CTN occurs via the production of ROS.

### 3.5. Effect of CTN on the Gene Expressions of KA Biosynthesis Genes

**Figure 6:**
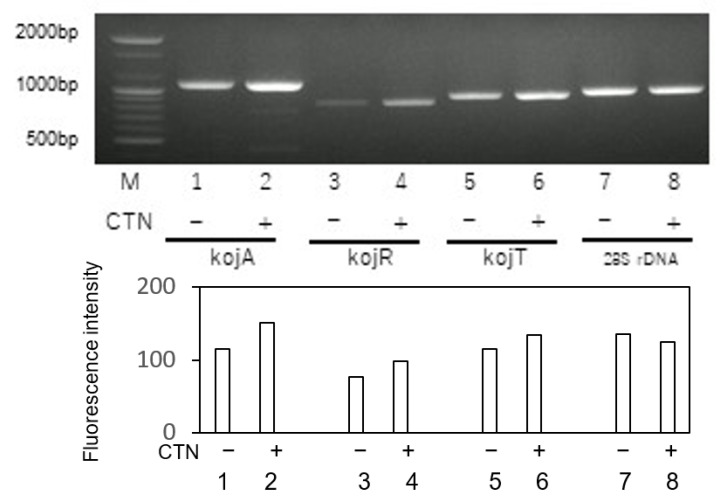
RT-PCR results for three KA biosynthesis genes. Total RNA was prepared from mycelia of *A. parasiticus* NIAH-26 which had been cultured in 200 μL of YES medium in the absence (lanes 1, 3, 5, 7) or the presence (2, 4, 6, 8) of 0.63 mM CTN in tip culture for 3 days, and the total RNA was isolated from the mycelial mat. RT-PCR was then performed for the genes *kojA* (lanes 1, 2; 1076 bp), *kojR* (lanes 3, 4; 831 bp), *kojT* (lanes 5, 6; 899 bp), and 28S rDNA (lanes 7, 8; 939 bp). Lane M: 100-bp ladder DNA marker (upper panel). An analysis using Image-J software showed that the expressions of the KA genes were increased, in contrast expression of the housekeeping 28S rDNA gene did not change (**lower** panel).

To further investigate the enhancement of KA production by CTN, we examined the expressions of KA biosynthesis genes in fungal mycelia that had been cultured in YES medium or YES medium supplemented with CTN. Although all of the KA biosynthesis genes *kojA*, *kojR*, and *kojT* were expressed in the absence of CTN, the expressions of all three were increased by the addition of CTN (Figure 6). These results suggested that the enhancement of KA production by CTN occurs at a transcription step of KA biosynthesis genes.

### 3.6. Effect of α-Tocopherol on Aflatoxin Production as Well as Fungal Growth

**Figure 7:**
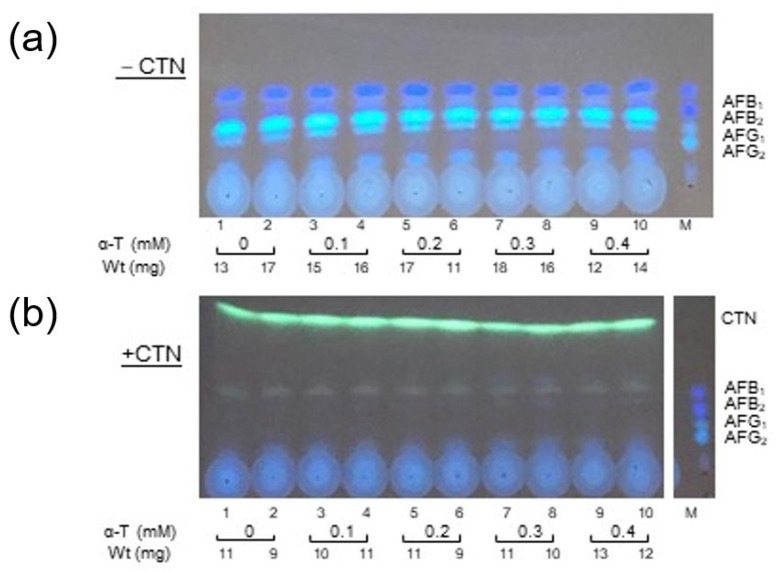
The effect of α-tocopherol on *A. parasiticus* NIAH-48B11 (AF^+^, KA^−^) and that of KA on *P. citrinum* NFRI-MI190. *A. parasiticus* 48B11 (AF^+^, KA^−^) was incubated in 200 μL of YES medium supplemented with various concentrations of α-tocopherol (α-T) in the absence (**a**) or presence (**b**) of 0.63 mM CTN for 3 days. The resulting medium (10 μL) was analyzed by TLC using solution D. The weight of the mycelia was measured. Experiments were done in duplicate.

*A. parasiticus* 48B11 was incubated in YES medium or YES medium supplemented with α-tocopherol concentrations from 0.1 mM to 0.4 mM (Figure 7). The production of AF was inhibited with CTN, and this inhibition was not recovered by the addition of these concentrations of α-tocopherol. This result suggested that ROS stress may not be involved in the inhibitory effect of CTN on the production of AF. In addition, although CTN inhibited the fungal growth of the 48B11 strain to approx. 67% of that without CTN, and the inhibition ratio was not significantly changed by the addition of 0.1–0.3 mM α-tocopherol. Although high concentration (0.4 mM) of α-tocopherol appeared to be different (Figure 7a,b), a more detailed study of the effects of α-tocopherol on AF production and the fungal growth is in progress in our laboratory.

### 3.7. Effect of KA on the Growth of P. Citrinum

**Figure 8:**
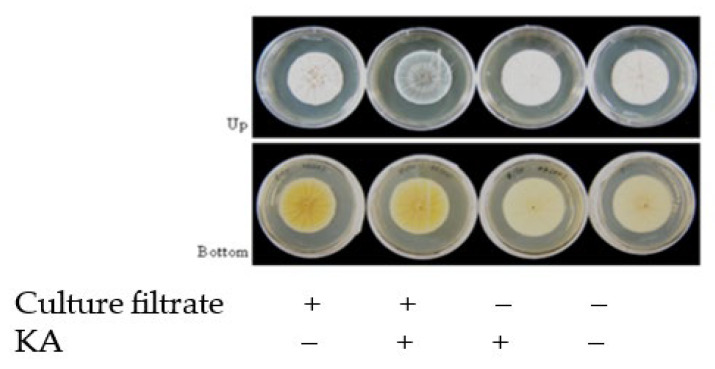
Effect of the culture filtrate of *P. citrinum* NFRI-MI190 and/or KA on growth of the NFRI-MI190. The NFRI-MI190 strain was cultured in the medium supplemented without or with either or both culture filtrate (containing CTN) of NFRI-MI190 and 100 mM KA in a 6 cm diameter plate for 3 days. The resulting colonies were observed from upper side (upper) or underside (lower) of the plate.

*P. citrinum* NFRI-MI190 was cultured in a GY agar plate supplemented (10-fold dilution) with the culture filtrate *P. citrinum* NFRI-190 and/or 100 mM KA for 4 days (Figure 8). KA did not affect the growth of *P. citrinum*, whereas the addition of KA slightly changed the color of the conidiospores of *P. citrinum* (Figure 8). This might suggest that *P. citrinum* is affected by *Aspergillus* species via the formation of KA.

## 4. Discussion

### 4.1. The Detoxication of CTN with KA to Form the CTN-KA Adduct

CTN is a mycotoxin that is harmful to various organisms and cell types. The detoxication of CTN has been extensively studied in experiments applying heat treatment and solvent extraction, among other methods. It was reported that heating CTN at 175 °C in the dry state or at 140 °C in an aqueous condition resulted in the detoxication of CTN, and the toxicity of CTN toward Hela cells was reported to be significantly reduced by heating at 130 °C in the presence of a small amount of water [17]. However, Trivedi et al. demonstrated that the heat treatment of CTN at 140°–150 °C caused the production of two substances, and among them citrinin H1 (a compound of two molecules of CTN) was confirmed to be more toxic than CTN [18], whereas Hirota et al, reported that citrinin H2 (a compound containing an opened pyran ring of CTN) was less toxic than CTN [19]. These results suggested that the use of high temperatures requires some effective control techniques to minimize the production of toxic substances from CTN.

In contrast, a solvent extraction method revealed that 91.6% of CTN was successfully removed from the fermented foods with *Monascus* species, and 79.5% of monacolin K, which has been widely used as a lipid-lowering drug, was also removed [20]. These findings indicate that the pre-establishment of the optimum conditions for a highly efficient and exclusive extraction of CTN is required for the practical uses of CTN. In addition, since solvent extraction requires equipment that is suitable for organic solvents and the extraction process, this method can be difficult to apply for practical use. The method using KA for the detoxification of CTN requires only the incubation of CTN and KA.

The formation of the CTN-KA adduct does not require high (>100 °C) temperatures. Incubation at 28 °C, 37 °C, or 50 °C was sufficient for the conversion of CTN to the CTN-KA adduct, and the CTN-KA adduct was a sole product of CTN and KA [12]. This method also does not require any special equipment, unlike the solvent extraction method. The method for the detoxification of CTN using KA described herein seems to be a simple, low-cost, safe, and practical method. We also expect that KA might be useful to detoxicate other harmful substances including other types of mycotoxins. We are now studying the adduct formation of other substances with KA in our laboratory.

KA has been used as an ingredient in cosmetics, i.e., for a whitening agent and a protective against UV light [21]. KA is also widely used clinically as an anti-fungal and anti-bacterial agent, painkiller and anti-inflammation drug, and cytotoxic agent; as an anti-melanosis agent, an anti-browning agent for fruits Xanthan gum, a precursor for flavor enhancers in the food industry field [22], a reagent for an ion determination substrate for comenic acid synthesis in the chemical industry, and as an insecticidal mycotoxin, chelating agent, and pesticides in agriculture [23]. The function of KA as an anti-leishmanial agent was recently reported [24], and a possible role for KA as a control reagent of a soybean disease has been described [25].

KA has been known to have anti-bacterial and anti-fungal effects on several genera of bacteria including *Agrobacterium*, *Bacillus*, *Chromobacterium*, and *Clostridium*, yeast and fungi, and more [26], indicating that KA can also exert adverse effects on organisms. However, relatively high concentrations of KA were necessary to obtain such effects; for example, 1.0 mg mL^−1^ (3 mM) KA inhibited the swarming of *Azospirillum* and *Proteus mirabilis* and 50 mM KA (7.1 mg mL^−1^) completely inhibited *S. sclerotiorum* symptoms in soybean [26]. Our present findings also demonstrated that a high concentration of KA (30 mM) was necessary to inhibit the growth of the bacteria (Figure 2a,b). In the cases of supplementation, the regulation value of CTN is relatively high (European Food Safety Authority [EFSA] value: 2000 μg/kg). Although our previous work showed that 1–1.3% of the CTN-KA adduct was decomposed to CTN after incubation at either 37 °C or 50 °C for 48 h. in aqueous condition [12], the resulting concentration seems to be very low. The consideration of the effects of KA on the health of animals and humans thus does not seem necessary.

*Aspergillus* species such as *A. oryzae*, *A. sojae*, and *A. awamori* have been used for the production of many Asian and Japanese food products. Japanese ‘koji,’ which is made from steamed rice with *A. oryzae,* has been used for the production of various types of fermented food such as miso paste, sake (rice wine), and shoyu (soy sauce), among others; the fermented foods have been reported to contain KA [10,27,28]. The presence of KA in food might thus provide some functions for protecting health from the toxicity of CTN.

### 4.2. Effects of CTN on KA-Producing Fungi

**Figure 9:**
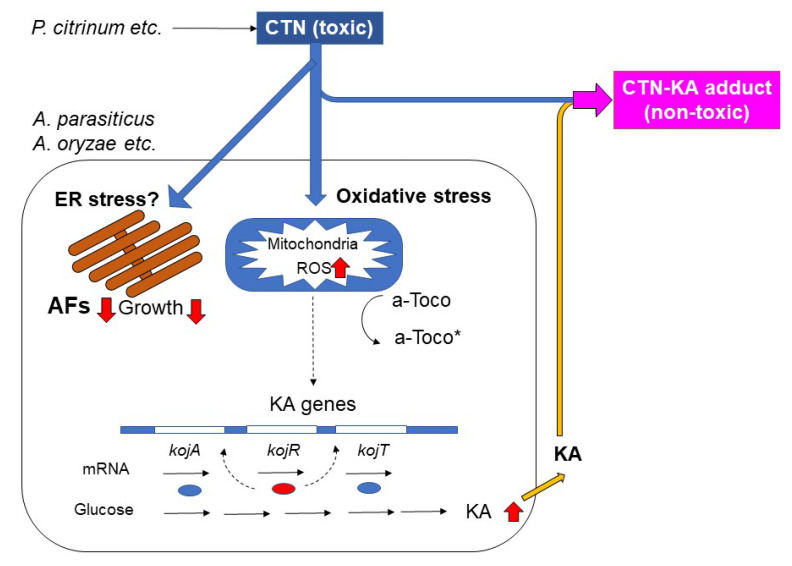
A postulated reaction mechanism of citrinin (CTN) on the productions of aflatoxin (AF) and kojic acid (KA) as well as the fungal growth of *A. parasiticus*.

Our results showed that CTN enhanced the production of KA in *A. parasiticus* as well as *A. oryzae* and that the enhancement of KA production by CTN was alleviated with α-tocopherol and BHA, which are ROS scavengers (Figure 5). Since CTN has been known to exert a variety of toxic effects on animals and human cells through oxidative stress by the production of ROS mainly in mitochondria [29,30,31,32,33], these results indicate that the ROS production caused by CTN may be involved in the enhancement of KA production. Figure 9 illustrates our postulated reaction mechanism of CTN on KA-producing fungi such as *A. parasiticus* and *A. oryzae*. We speculate that the transcription of the KA biosynthesis genes *kojA*, *kojR*, and *kojT* was caused via oxidative stress due to the production of ROS by CTN (Figure 6 and Figure 8). Even in the absence of CTN, a certain amount of KA was produced (Figure 5a–d), suggesting that a small amount of ROS might be endogenously produced. Our present findings also demonstrated that compared to the KA-producing strains, the KA-non-producing mutants or strains were more susceptible to CTN. These results indicate that KA-producing fungi such as *A. flavus* and *A. oryzae* may protect themselves from the toxic effect of CTN by a conversion of toxic CTN to the non-toxic CTN-KA adduct with KA, the production of which was further enhanced with CTN (Figure 3 and Figure 4).

We observed that CTN inhibited both the AF production and the fungal growth of *A. parasiticus* (Figure 3 and Figure 6); however, unlike the KA production, the inhibition of AF production as well as that of fungal growth was not clearly alleviated by α-tocopherol (Figure 7). A mechanism other than oxidative stress may be involved in these phenomena. Wu et al. recently reported that CTN-induced hepatotoxicity in mice was regulated mainly via Ca^2+^/endoplasmic reticulum (ER) stress, which is caused by alterations in Ca^2+^ homeostasis and an accumulation of misfolded proteins in the ER [5,34]. We suspect that Ca^2+^/ER stress might be involved in the inhibitions of AF production and fungal growth with CTN (Figure 9), but the mechanisms underlying these inhibitions remain to be clarified.

*Aspergillus* and *Penicillium* species are common in moderate-temperature regions, and fungi belonging to both of these genera are often detected in the same environments (e.g., soils and plants) [12]. We observed that AFs (Yabe, personal data) and KA (Figure 8) did not affect the growth of *P. citrinum*, whereas the addition of KA slightly changed the color of the conidiospores of *P. citrinum*, suggesting that *P. citrinum* might also be affected by *Aspergillus* species via the formation of KA. It might be possible that *Aspergillus* species and *Penicillium* species interact (or communicate) with each other via the formation of their secondary metabolites. We are examining this possibility in our laboratory.

## Data Availability

Not applicable.
